# Tuning of human NK cells by endogenous HLA-C expression

**DOI:** 10.1007/s00251-020-01161-x

**Published:** 2020-03-26

**Authors:** Frederick J. Goodson-Gregg, Stacey A. Krepel, Stephen K. Anderson

**Affiliations:** 1 Laboratory of Cancer Immunometabolism, Center for Cancer Research, National Cancer Institute, Frederick, MD 21702 USA; 2 Basic Science Program, Frederick National Laboratory for Cancer Research, Frederick, MD 21702 USA

**Keywords:** HLA-C, NK, NK promoter, NK education, Licensing, Arming

## Abstract

NK cells are primarily responsible for detecting malignant or pathogen-infected cells, and their function is influenced both by stress-associated activating signals and opposing inhibitory signals from receptors that recognize self MHC. The receptors that produce this inhibitory signal shift from the NKG2A:HLA-E system to that of KIR:HLA as the NK cells mature. This maturation is associated with an increase in lytic activity, as well as an increase in HLA-C protein levels controlled by the NK-specific *HLA-C* promoter, NK-Pro. We propose that modulation of the translatability of *HLA-C* transcripts in NK cells constitutes an evolutionary mechanism to control *cis* inhibitory signaling by HLA-C, which fine tunes NK cell activity. Furthermore, the high degree of variability in KIR receptor affinity for HLA alleles, as well as the variable expression levels of both KIR and HLA, suggest an evolutionary requirement for the tuning of NK lytic activity. Various data have demonstrated that mature NK cells may gain or lose lytic activity when placed in different environments. This indicates that NK cell activity may be more a function of constant tuning by inhibitory signals, rather than a static, irreversible “license to kill” granted to mature NK cells. Inhibitory signaling controls the filling of the cytolytic granule reservoir, which becomes depleted if there are insufficient inhibitory signals, leading to a hyporesponsive NK cell. We propose a novel model for the tuning of human NK cell activity via *cis* interactions in the context of recent findings on the mechanism of NK education.

## Introduction

Natural killer (NK) cells are innate lymphocytes possessing a variety of activating receptors that detect cells undergoing stress, malignant transformation, or the presence of pathogens (Arase et al. 2002; Raulet et al. 2017; Barrow et al. 2019). The signals transmitted by these activating receptors are opposed by inhibitory signals generated by receptors that recognize MHC proteins (Karlhofer et al. 1992; Colonna and Samaridis 1995; McQueen and Parham 2002). Human NK cells are defined as lymphocytes that express neural cell adhesion molecule 1 (*NCAM1*/CD56), and lack surface expression of the T-cell receptor-associated complex epsilon chain (*CD3E*/CD3) (Lanier et al. 1986).

Human NK cells are divided into early and late developmental stages based on the level of CD56 expression, either CD56^bright^ or CD56^dim^, respectively (Freud and Caligiuri 2006). The CD56^dim^ subset generally accounts for greater than 80% of peripheral blood NK cells. CD56^dim^ NK cells express killer cell immunoglobulin-like receptors (KIR), cytolytic granules containing perforin (*PRF1*) and granzyme B (*GZMB*), and high surface levels of the low-affinity immunoglobulin gamma Fc region receptor III-A (*FCGR3A*/CD16), enabling the killing of antibody-coated target cells. Although the less mature CD56^bright^ cells are a minor component of the peripheral blood NK population, they are the major subset in secondary lymphoid tissues (Ferlazzo et al. 2004). CD56^bright^ NK cells express lower resting levels of cytotoxic effector proteins, are generally CD16 negative, and use the CD94/NKG2A receptor complex (*KLRD1*/*KLRC1*) to achieve self-tolerance, as KIR expression is only found in the CD56^dim^ subset. CD56^bright^ NK cells are also potent cytokine producers (IFN-γ, TNF, GM-CSF) when stimulated with cytokine combinations; however, they show limited antitumor activity ex vivo (Fehniger et al. 1999).

NKG2A is the major inhibitory MHC receptor expressed by immature CD56^bright^ NK cells, and it recognizes the HLA-E molecule (Lee et al. 1998b). This is a highly conserved interaction, since HLA-E has few alleles and it presents the HLA leader peptide (Lee et al. 1998a; Parham et al. 2012). As NK cells mature, NKG2A expression is replaced by the variegated expression of KIR that recognize specific HLA-A, HLA-B, or HLA-C alleles (Béziat et al. 2010; Björkström et al. 2010).

**Fig. 1:**
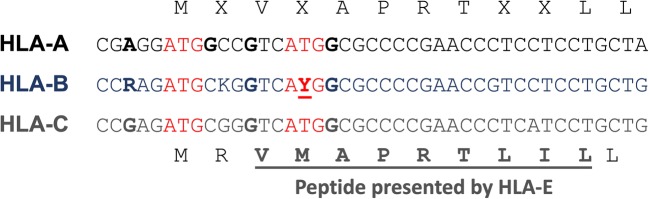
Evolution of the HLA-B and HLA-C leader peptide to decrease its binding to HLA-E. The DNA sequence of the translation initiation region of the *HLA-A*, *HLA-B*, and *HLA-C* genes is shown. Competing ATG elements are shown in red, and flanking nucleotides that enhance translation initiation are shown in bold. The dimorphic nucleotide in *HLA-B* that changes the fourth codon from ATG (methionine) to ACG (threonine) is indicated by the underlined bold Y. The consensus amino acid sequence of the leader region is shown above with variable amino acids indicated by X, and the HLA-C leader is shown below with the peptide presented by HLA-E underlined in bold

A comparison of the leader peptide of the HLA-A, HLA-B, and HLA-C proteins reveals an interesting feature that may reflect an evolutionary process whereby KIR:HLA-B/HLA-C interactions are supplanting NKG2A:HLA-E interactions to control NK activity. The *HLA-A* gene possesses two closely-spaced ATG codons that both conform to the optimal context for translation initiation: a purine at − 3 and a guanine at + 4 (Fig. 1) (Kozak 1986). In this context, the first ATG would be dominant, as demonstrated by Kozak (Kozak 2005). However, in the *HLA-B* and *HLA-C* genes, the guanine at + 4 of the first ATG has been replaced by a cytosine residue, which would allow for enhanced initiation at the downstream ATG. This is significant, as the leader peptide presented by HLA-E begins at the valine residue that precedes the second methionine. Therefore, the HLA-B and -C proteins produced by initiation at the downstream ATG would not provide ligands for HLA-E. Furthermore, the *HLA-B* gene contains a dimorphism that replaces the second ATG with ACG (threonine), resulting in a leader peptide that cannot be presented by HLA-E. HLA-B alleles possessing a leader peptide that can be presented by HLA-E typically do not contain the Bw4 epitope recognized by KIR3DL1 (Litwin et al. 1994; Lutz 2014), while those alleles that have leader peptides that cannot be loaded into HLA-E tend to possess the Bw4 epitope, indicating a shift from alleles that are recognized via NKG2A to those seen by KIR3DL1 (Horowitz et al. 2016).

All HLA-C alleles are recognized by at least one KIR, whereas less than half of the HLA-A or -B alleles are KIR ligands (Norman et al. 2007). KIR gene expression is activated by a probabilistic mechanism, and the majority of NK cells (~ 80%) express from 1 to 3 KIR (Valiante et al. 1997; Li et al. 2008). These NK cells are specialized, as their inhibitory receptors recognize a subset of HLA alleles, enhancing identification of aberrant cells which have lost or downregulated expression of a single HLA allele. MHC-C is the most recently evolved MHC class I, and is found only in humans and great apes, along with the appearance of the MHC-C-specific KIR: KIR2DL1; KIR2DL2; KIR2DL3 (Guethlein et al. 2007). Dimorphism at position 80 in the α1 domain of HLA-C defines two mutually exclusive epitopes, C1 (asparagine 80) and C2 (lysine 80), which are recognized by different KIR (Winter and Long 1997). KIR2DL1 recognizes HLA-C alleles possessing the C2 supratype, while KIR2DL2/L3 recognize the C1 group of HLA-C alleles.

The appearance of the *MHC-C* gene in the genome of hominid primates has been linked with a greater invasion of placental trophoblasts into maternal decidual tissue (Moffett and Colucci 2015). NK cells are the dominant lymphocyte population in the decidua, and they interact directly with invading trophoblasts, which lack expression of HLA-A and HLA-B but express HLA-C together with HLA-E, HLA-F, and HLA-G, indicating a potential role for KIR:HLA-C interactions in the regulation of trophoblast invasion (Redman et al. 1984; Ishitani et al. 2003; Apps et al. 2009; Hackmon et al. 2017; Moffett et al. 2017). Specific combinations of maternal *KIR* and fetal *HLA-C* alleles are associated with either preeclampsia and low birth weight, or obstructed labor, indicating that the balance of inhibitory/activating receptor interactions in NK cells can affect pregnancy outcomes (Hiby et al. 2004, 2010, 2014).

## Licensing, education, and tuning via inhibitory signaling

The activity of natural killer cells, as measured by either release of IFN-γ or cytolytic granules, is enhanced by the presence of inhibitory receptors that recognize MHC ligands expressed by the host. This was first observed in the murine system and was described as “licensing” of the NK cell (Kim et al. 2005). A similar phenomenon was subsequently reported in the human system and has been referred to as “NK cell education” (Anfossi et al. 2006; Kim et al. 2008; Yawata et al. 2008). Individual NK cells stochastically express self MHC I-specific inhibitory receptors, and the affinity of inhibitory receptors for MHC ligands varies; thus, NK cells are exposed to differing degrees of inhibition, depending on their inhibitory receptor repertoire and the MHC molecules that are expressed in their environment (Held and Kunz 1998; Parham 2006). NK cells expressing multiple self-specific inhibitory receptors are the most responsive subset. As the number of inhibitory receptors decreases, the cells become less responsive. Similar results were generated in the analysis of human and mouse NK cells, leading to the concept that the NK response is tuned through the integration of multiple inhibitory and stimulatory signals (Yu et al. 2007; Fauriat et al. 2010; Béziat et al. 2013). Progressively decreasing the number of MHC molecules expressed by mice, using gene knockouts and transgenic animals resulted in progressively lower responsiveness of the NK cells in the animals (Brodin et al. 2009b). Conversely, human NK cells lacking a self-ligand for a specific KIR gained reactivity following transfer to mice expressing the HLA ligand for that KIR (Boudreau et al. 2016). Collectively, these findings suggest that the responsiveness of NK cells increases in proportion to the amount of inhibitory signaling the cell receives.

These data led to the analogy of a “rheostat” that governs NK cell activity, which was proposed to explain the observation that the magnitude of the murine NK response varies, rather than reflecting only responsive or hyporesponsive states (Brodin et al. 2009a; Joncker et al. 2009). For an electrical system, a rheostat is a variable resistor that controls current. The analogy with respect to NK cell function is that target cell activation of NK cells is opposed by the resistance generated from the inhibitory receptors. However, it is important to consider both the lytic potential and the threshold of activation together, as increased resistance to activation should also lead to an increased stored capacity which would result in an enhanced capacity to mediate serial killing (Prager et al. 2019). The acquisition of lytic activity associated with the mature CD56^dim^ stage of NK cell differentiation can also be viewed as an on/off switch that is subsequently controlled by a rheostat.

It is important to note that differences in the activity of mature NK cells attributed to education/tuning are not due to changes in gene expression, but rather are the result of differences in signaling. Examination of gene expression in beta-2 microglobulin (β2m) or K^b^D^b^ knockouts compared with control C57BL/6J mice revealed no functionally significant change in gene expression, but rather a change in the membrane compartmentalization of activating and inhibitory receptors (Guia et al. 2011). Furthermore, RNAseq of human CD56^dim^ NK cells also showed no significant difference between educated and uneducated subsets. Gene expression patterns were highly correlated when subsets with a KIR receptor recognizing self MHC were compared with NK cells expressing KIR that lacked a ligand (Goodridge et al. 2019). In contrast, a comparison of CD56^bright^ versus CD56^dim^ NK cells showed that differentiation and KIR acquisition were associated with increased transcription of granzyme B and several other genes known to be involved in effector function (Goodridge et al. 2019). Therefore, the differentiation of NK cells from the CD56^bright^ to CD56^dim^ stage turns on the lytic machinery that is then subject to tuning by KIR inhibitory receptors that are expressed at this stage. A recent study has revealed functional differences in the behavior of educated NK cells by single-cell imaging of NK cells possessing only a single inhibitory receptor (CD56^dim^/KIR^−^/NKG2A^+^) versus NK cells lacking all inhibitory receptors (CD56^dim^/KIR^−^/NKG2A^−^). NKG2A-expressing NK cells displayed increased migration, made more contacts with target cells, and killed targets more frequently than receptor-negative cells. NK cells capable of serial killing were primarily found within the NKG2A^+^ cell population, indicating an increase in stored lytic capacity (Forslund et al. 2015).

The effect of removing inhibitory signals from NK cells at equilibrium should result in a lower threshold of activation and subsequent depletion of the cytolytic reservoir, defined as the reserve of mature cytolytic granules in an NK cell. This loss of responsiveness has been observed when murine NK cells are moved to an environment that lacks MHC ligands. When human NK cells lacking a self-ligand for a specific KIR were transferred to transgenic mice that expressed the HLA ligand for that KIR, those NK cells gained reactivity (Boudreau et al. 2016). Target cell HLA-C has been shown to be transferred to the NK cell plasma membrane and cytoplasm in a KIR-dependent manner. Only HLA-C alleles that are recognized by KIR2DL1 (C2 group) are transferred to KIR2DL1-expressing NK cells and this process depends on NK cell ATP (Carlin et al. 2001). While *cis* interactions in murine NK cells have been clearly demonstrated, no direct evidence of the physical *cis* HLA:KIR interaction in human NK cells has been identified. Surprisingly, human NK cells that possessed a KIR:HLA ligand pair retained their activity when transferred to mice lacking the ligand, indicating an NK cell intrinsic, or *cis* effect of HLA expression. Additionally, knock-down of HLA within the human NK cells decreased their function, thus indicating the importance of *cis* interactions for maintaining their lytic potential (Boudreau et al. 2016). Allelic variation in the level of HLA-C cell surface expression has been inversely correlated with lytic activity, providing further evidence for the *cis* effect of NK cell HLA expression (Li et al. 2018). The inability of murine NK cells to retain their responsiveness when placed in a mouse lacking MHC ligands—contradictory to the maintained human NK activity when similarly transferred—suggests that the *cis*-signaling mechanisms operating in the human and mouse systems are distinct. Murine NK cells possess both *cis* and *trans* interactions, and it appears both are necessary for NK activity and the generation of a normal MHC receptor repertoire (Bessoles et al., 2013). We speculate that human NK cells maintain activity via an either/or mechanism, rather than requiring both *cis* and *trans* to function properly.

The murine Ly49 molecules contain a flexible stalk that permits interaction with MHC in *cis* on the NK cell surface, and this interaction has been shown to be required for the licensing of mouse NK cells (Doucey et al. 2004; Bessoles et al. 2013). KIR molecules lack flexible stalks, and are not expected to engage in *cis* interactions at the cell surface (Held and Mariuzza 2008). Recently, however, there have been several lines of investigation examining *cis* signaling in NK cells that suggest alternative means for such an interaction to take place. For example, LILRB1 has been shown to interact in *cis* with HLA class I, despite lacking a flexible stalk, challenging the notion that for a *cis* interaction to occur, it must do so similarly to the flexible Ly49 molecule (Li et al. 2013). Furthermore, we speculate that the endosomal compartment may provide an environment for HLA-C:KIR *cis* interaction and inhibitory signaling to occur within NK cells. Surface class I MHC are continually endocytosed, and are either degraded, or recycled back to the plasma membrane (Donaldson and Williams 2009). We speculate that both KIR and their ligands are continually brought into close proximity within endosomes, offering a more favorable geometry for their interaction and subsequent inhibitory signaling. Consequently, the higher levels of surface HLA-C generated by the NK-Pro would be expected to contribute to greater *cis* inhibitory signaling in the NK cell endosomal compartment. Lastly, in line with the findings that cell-intrinsic class I expression is required to retain effector potential, it is possible that the LILRB1 receptor contributes to *cis* inhibition of NK cells, due to its broad reactivity for HLA class I molecules. However, the magnitude of inhibition by LILRB1 is generally lower than KIR, and the level of LILRB1 inhibition associated with HLA-C binding is marginal when compared to HLA-A, HLA-B, and HLA-G binding (Vitale et al. 1999). While there is an increasing body of evidence for the importance of *cis* signaling for lytic activity and maintenance of the cytolytic reservoir, further research is necessary to clarify the mechanism of *cis* inhibition and its role in arming/licensing of human NK cells.

## Fine tuning of the KIR:HLA inhibitory signal

Although the response to pathogens is a major force driving the diversification of HLA and KIR alleles, the importance of an appropriate level of KIR/HLA interaction for preventing excessive immune activation and for successful reproduction places a strong selective pressure on this system that requires tuning of the inhibitory signal.

The evolution of a finely balanced system is reflected in the high degree of variability in receptor:ligand affinities and expression levels of KIR/HLA alleles. The affinity of a given KIR2DL1 allele for different HLA-C2 alleles varies, as does the affinity of a given HLA-C2 allele for individual KIR2DL1 alleles. The same variation is present for KIR2DL2/2DL3 recognition of the C1 group of alleles (Hilton et al. 2015a). There is well-documented variation in cell surface expression levels of KIR and HLA as well (Yawata et al. 2006). The primary role of HLA-C in tuning NK cell activity is also supported by its low expression level when compared with the ten-fold higher level of HLA-A or HLA-B on most cell types (Apps et al. 2015). This lower level of HLA-C expression may be necessary in order to achieve a tunable threshold of inhibitory signaling by KIR. Moreover, HLA-C has a less efficient peptide-binding pocket and weaker association with β2m that reduces the rate of assembly and export of HLA-C:peptide complexes and their stability on the cell surface (Güssow et al. 1987; Neisig et al. 1998). If cells are infected by pathogens that downregulate HLA to avoid T-cell recognition, this lower expression level of HLA-C provides an increased likelihood of a significant loss of inhibitory KIR signaling when downregulated. The core *HLA-C* promoter lacks NF-κB-binding sites, and this results in decreased promoter activity and a lack of responsiveness to TNF-mediated induction of transcription, thus maintaining low levels of HLA-C expression at sites of inflammation (Anderson 2018). Tuning of the KIR:HLA-C interaction is also reflected in allelic variation of the cell surface level of HLA-C expression, which has been associated with differential outcomes in HIV infection (Apps et al. 2013). Multiple mechanisms have been described that contribute to variation in cell surface levels of HLA-C, including promoter/enhancer polymorphisms, variation in 3′-UTR miRNA binding sites, and efficiency of peptide binding (Kulkarni et al. 2011; Vince et al. 2016; Kaur et al. 2017). The higher cell surface expression of the HLA-C*05 and HLA-C*06 alleles has been associated with differences in the peptide-binding domain of these alleles (Kaur et al. 2017; Goodson-Gregg et al. 2020), consistent with the constraints placed upon HLA-C expression by their inefficient assembly and export.

The MHC environment is a powerful determinant of NK cell function. For example, the strength of the KIR:HLA-C interaction appears to be predictive of the magnitude of the missing-self response. NK cells expressing only KIR2DL3 have been shown to mount a stronger missing-self response in donors carrying the more strongly interacting HLA-C*07 (C1) when compared to the weaker interaction when HLA-C*1402 (C1) is present (Yawata et al. 2008). Thus, in contrast to the view of a fixed NK cell lytic potential, the reality appears closer to a constantly “tuned” NK cell that has its cytolytic threshold determined by inhibitory signals. It may be more informative to consider education as an all-or-none digital event that leads to NK cell maturation, and to interpret differences in NK activity associated with the strength of receptor:ligand interactions as an analog mechanism, or rheostat, that tunes the responsiveness of mature cytolytic NK cells (Brodin et al. 2009a; Joncker et al. 2009). From this perspective, NK cells would be classified as either mature (CD56^dim^ cells expressing KIR receptors) or immature (CD56^bright^ cells lacking KIR) with variable NKG2A expression, and differences in the magnitude of the missing-self response in mature cytolytic NK cells would be attributed to variation in the strength of inhibitory signaling.

## NK-specific *HLA-C* transcripts

The recent discovery of an NK-specific *HLA-C* promoter (Li et al. 2018), which produces a wide array of differentially spliced *HLA-C* mRNAs with distinct translational efficiencies, points to an important role for endogenous HLA-C expression in NK cells and indicates the emergence of an NK-specific HLA-C-tuning mechanism. Although upstream *HLA-A* transcripts have been identified in a macrophage cDNA library, there only appears to be a single mRNA isoform generated (Li et al. 2018). The NK-specific transcripts of *HLA-C* contain 3 non-coding exons that vary in size due to the presence of alternative splice donor and acceptor elements, some of which are allele-specific. These exons have been named −1a, −1b, and −1c. The first coding exon (exon 1), located adjacent to the core *HLA-C* promoter, also varies in size when transcription is initiated from the upstream NK-specific promoter: this is due to the differential use of multiple exon 1 splice acceptors. However, only one alternative splicing event in the 5′-UTR affects the HLA-C open reading frame: in a subset of NK promoter transcripts, exon −1c is spliced to exon 2, thus skipping the initiation codon in exon 1 and resulting in an untranslatable HLA-C message. This untranslatable message is more abundant in immature NK cells, and decreased exon 1 skipping is associated with increased HLA-C expression as NK cells mature. Retention of introns 1 and 2 has also been observed in bone marrow and spleen, providing an additional mechanism that would prevent protein expression from either the proximal or NK-specific *HLA-C* promoter transcripts (Li et al. 2018).

**Fig. 2:**
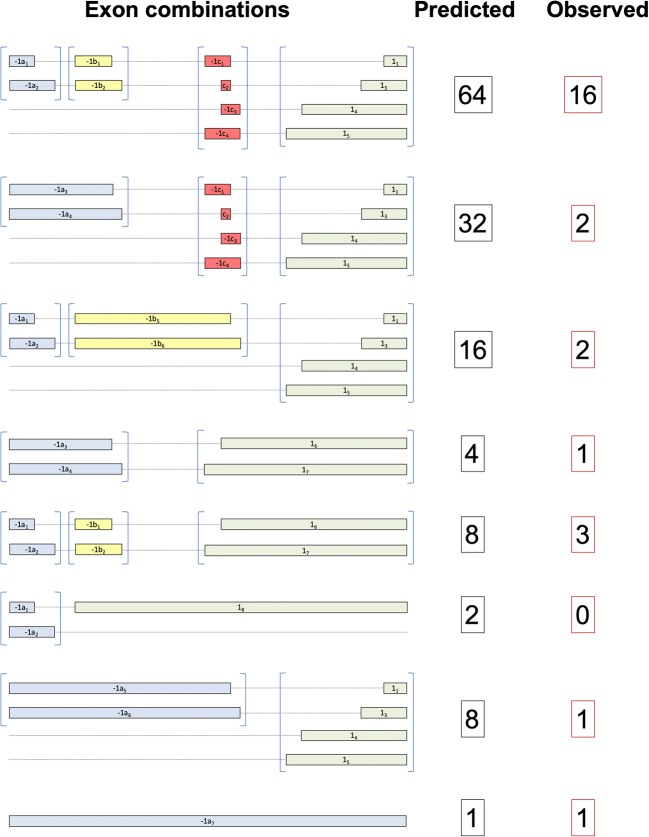
The predicted versus observed splice variants of the HLA-C*06 gene. All possible combinations of the alternative exons observed for the HLA-C*06 allele are listed, beginning with a group depicting the 64 combinations predicted by the separate inclusion of each of the four possible exons: −1a (blue); −1b (yellow); −1c (red); exon 1 (green). Exon groups are contained within parentheses. Subsequent groups indicate splice forms resulting from varying retention of the three introns that separate these exons. The predicted number of exon combinations is shown in the black box next to each group, and the observed number of splice forms is shown in the red box

Seven −1a exon variants have been observed, four allele-specific −1b exons, four −1c exons, and seven exon 1 variants, one of which is allele-specific. Therefore, a large number of distinct 5′-UTR sequences with different combinations of these exons could be generated. However, due to the overlap of the larger exon −1a and exon 1 splice variants with the −1b and −1c exons, and the presence of allele-specific exons, the number of possible exon splicing combinations is 135 for the HLA-C*06 allele alone, and of these only 25 have been observed to date (Fig. 2).

There are currently 42 distinct full-length *HLA-C* splice forms deposited in GenBank, however, direct analysis of the 5′-UTR region by RT-PCR indicates that many more splice forms exist. The extreme diversity of the NK-specific *HLA-C* transcripts is remarkable, and the observation of distinct patterns of transcripts in NK cells from different tissues suggests the evolution of a mechanism to fine-tune HLA-C expression in NK cells in response to distinct environmental stimuli (Li et al. 2018).

The hypothesis that alternative 5′-UTR content arose to fine-tune HLA-C expression levels in developing NK cells in different tissues, predicts that the translatability of alternatively spliced *HLA-C* mRNAs varies and is controlled by alternative exon usage. Initial analyses indicated a linear relationship between increased 5′-UTR size and decreased levels of protein expression (Li et al. 2018). A more detailed analysis of a large panel of differentially spliced *HLA-C* mRNAs has revealed that the presence of competing AUG start codons also contributes to the variable translatability of *HLA-C* mRNA isoforms (Goodson-Gregg et al. 2020). The exon −1c splice donor and exon 1 splice acceptor combination determines if a competing AUG in exon −1c produces a short open reading frame upstream of the *HLA-C* initiation codon, or if it generates a longer open reading frame that overlaps the *HLA-C* AUG and prevents its use. Larger exon 1 isoforms (exons 1_5_–1_7_) also contain a 42 amino acid open reading frame that inhibits translation. However, increased length alone is not sufficient to decrease translatability, as the larger exon −1a_3_ isoform increases expression, likely due to the addition of an A/T-rich sequence at the 5′ end of the mRNA, weakening secondary structure and facilitating ribosome entry and scanning.

## Tissue specificity of *HLA-C* mRNA isoforms

The patterns of *HLA-C* NK promoter transcripts have been analyzed in bone marrow, spleen, and peripheral blood, revealing extensive differences in exon usage between these tissues (Li et al. 2018). Some of these differences may reflect the developmental stage of the NK cell, such as an abundance of poorly translatable and untranslatable isoforms in bone marrow and spleen due to the presence of immature NK cells. However, an RT-PCR comparison of 5′-UTR *HLA-C* mRNA isoforms in peripheral blood from donors with matched *HLA-C* alleles revealed significant differences in the minor splice forms observed (Goodson-Gregg et al. 2020). These differences potentially represent the presence of tissue-resident NK cells, which have entered the blood due to inflammation or stress occurring in a particular organ. A thorough characterization of *HLA-C* splice forms in multiple tissues may provide a “splicing profile,” capable of determining the tissue of origin of circulating NK cells. The possibility that NK cells possess distinct *HLA-C* splice forms that reflect the tissue in which they reside could potentially lead to a method for evaluating NK cell dynamics in health and disease. Notably, extensive alternative splicing has been observed for the *KIR* and *LILRB1* genes, resulting in the production of alternative protein isoforms (Bruijnesteijn et al. 2018; Jones et al. 2009). Whether these alternative splice forms are regulated in a developmental or tissue-specific manner remains to be determined.

## NK-Pro transcripts are absent in some *HLA-C* alleles

The emergence of an elaborate mechanism to control HLA-C levels within NK cells suggests an important role in their development or function. However, there are five *HLA-C* alleles that do not express NK-Pro transcripts due to a polymorphism in a key Ets-binding site in the promoter (Li et al. 2018). Two alleles are of the C1 group (C*07, C*08) and three belong to the C2 group (C*02, C*05, C*17). The frequency of individuals that possess HLA-C alleles lacking a functional NK-Pro is approximately 50% (http://www.allelefrequencies.net/default.asp). Upregulation of HLA-C expression in NK cells by the NK-Pro could have evolved in order to compensate for differing levels of affinity of the HLA-C alleles for their cognate KIR. This would predict that an HLA-C allele with a high affinity for its cognate receptor would be more likely to lack NK-Pro transcripts. Although an initial study demonstrated significantly higher binding of the NK-Pro deficient HLA-C*02 and HLA-C*05 alleles to KIR2DL1 than HLA-C*06 (Moesta et al. 2008), subsequent studies did not (Hilton et al. 2012, 2015b). Furthermore, the non-upregulated HLA-C*07 and HLA-C*08 alleles did not exhibit higher binding to KIR2DL2 or KIR2DL3 than other C1 group alleles that have an active NK-Pro and are expressed at higher levels on mature NK cells (Moesta et al. 2008; Hilton et al. 2012, 2015b; Frazier et al. 2013). Therefore, it is more likely that the modulation of HLA-C levels by the presence or absence of alternatively spliced NK-Pro transcripts occurs in order to control the level of HLA-C expression in a developmental and tissue-specific manner, as evidenced by the increase in translatable NK-Pro transcripts in mature NK cells (Li et al. 2018).

If the system of HLA-C regulation in NK cells evolved to provide a specialized tuning system that acts to modulate NK cell reactivity after they have become “licensed killers,” and increase the threshold of activation so that a stronger signal is required to trigger killing, then the predicted effect of NK-Pro presence or absence would be differences in the level of self-reactivity. Since NK cells have been shown to limit the magnitude of the anti-viral T-and B-cell responses (Waggoner et al. 2016), the outcome of viral infections, especially the degree to which NK cells kill activated CD4 cells, could be modulated by variation in the endogenous HLA-C levels mediated by the NK-Pro. It may be informative to look at the outcome of viral infections in individuals lacking the *HLA-C* NK-Pro versus those capable of upregulating HLA-C in mature NK cells. In addition, since HLA-C is also upregulated in KIR-expressing decidual NK cells, there may be differences in pregnancy outcomes associated with the presence or absence of NK-Pro transcripts.

We propose that the variation in expression level of HLA-C mediated by alternative splicing of NK-Pro transcripts during the process of NK cell education and differentiation serve to modulate the selectivity and killing potential of NK cells during their development. First, it is worth deconstructing the concept of licensing/education of an NK cell versus the tuning of lytic potential in mature NK cells. Immature CD56^bright^ NK cells are not innately cytolytic; however, naive and inhibitory receptor-deficient NK cells can have an effector phenotype when stimulated by cytokines to express perforin and granzymes (Fehniger et al. 2007; Björkström et al. 2010; Romee et al. 2012). This is of particular importance, as NK cells can possess an effector phenotype regardless of education status. NK cells that lack self-specific receptors are hyporesponsive (Fernandez et al. 2005; Kim et al. 2005). However, even NK cells that are hyporesponsive can still respond to cytokines. In the murine system, 10–13% of the NK cells lack MHC-specific receptors and are hyporesponsive; yet, they remain capable of responding to infection normally and secreting IFN-γ (Fernandez et al. 2005). While proper exposure to activating and inhibitory signals are required for licensing/education and spontaneous effector phenotype, these cells can still lose their activity if not exposed continuously to proper environmental stimuli. Murine NK cells moved to an MHC-deficient mouse lose functional responsiveness within 4 days (Elliott et al. 2010; Joncker et al. 2010).

As NK progenitor cells differentiate into immature NK cells in the bone marrow, the NK-Pro becomes active. At this stage, the transcripts originating from this promoter are largely untranslatable in the immature cell, owing to the majority of splice forms skipping exon 1, or retaining introns 1 or 2. Moreover, as NK cells mature and gain lytic activity, translatable NK-Pro transcripts become more abundant (Li et al. 2018). A comparison of individuals possessing an intact Ets site in the NK-Pro to those that do not, reveals a nearly two-fold increase in total cell surface HLA-C protein on CD56^dim^ NK cells due to a functional NK-Pro (Li et al. 2018). Taken together, these observations raise an interesting question as to the functional advantage of delaying this increase in protein expression until the cell is fully mature. The functional effect of the switch from low endogenous HLA-C expression in immature NK cells to high expression in mature cytolytic NK may indicate a transition from education by stromal cells to attenuation of the missing-self response by endogenous HLA-C as NK cells mature.

## Concluding remarks

**Fig. 3:**
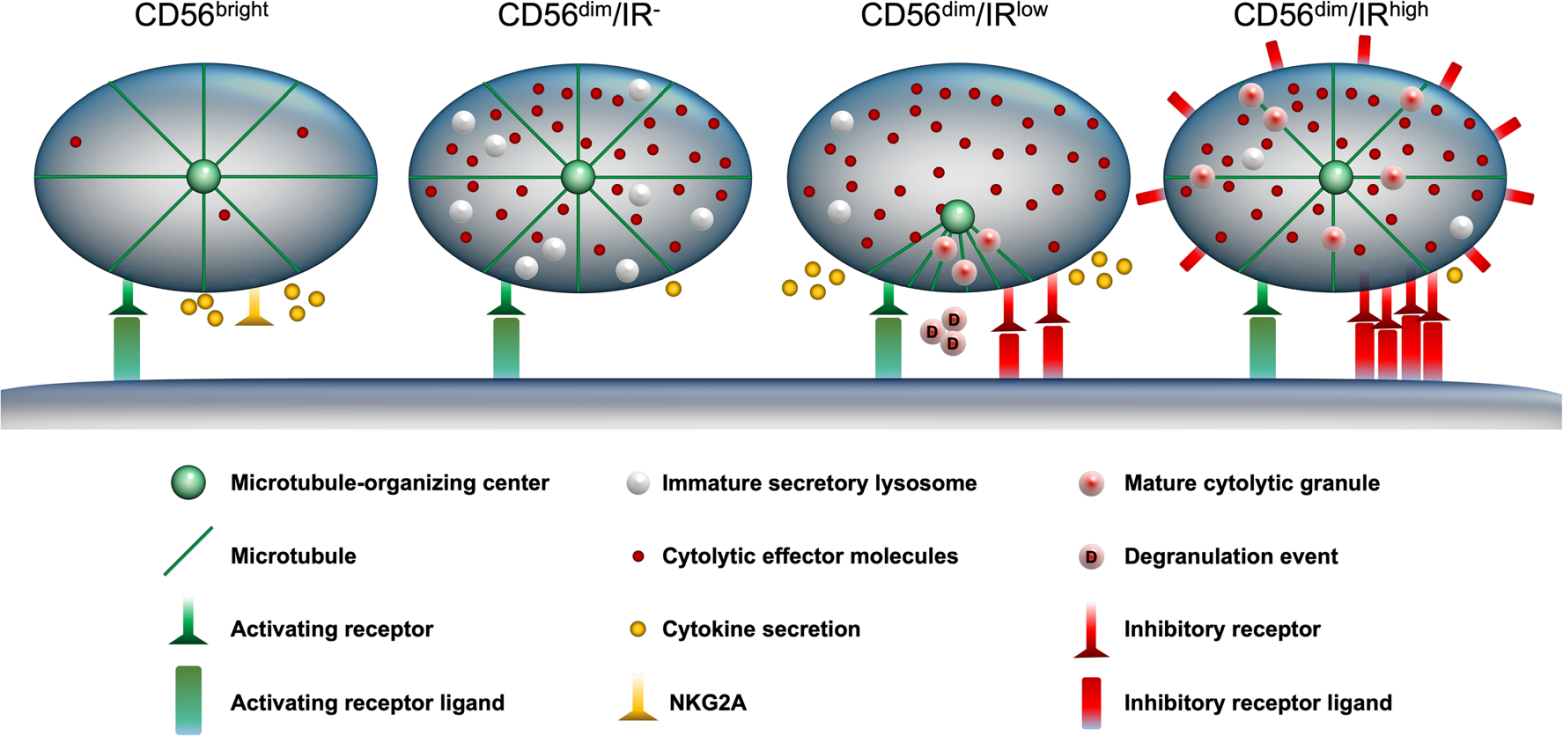
Model depicting distinct states of human NK cell development/activity. Four states of NK cell differentiation are shown, illustrating the differences in receptor expression and their impact on functional competency. CD56^bright^ NK cells have low cytolytic activity, express NKG2A, and secret cytokines when activated. CD56^dim^ inhibitory receptor-negative (IR^−^) cells have upregulated cytolytic effector molecules but are unable to generate mature lytic granules. CD56^dim^ cells expressing inhibitory receptors at a low level (IR^low^) can make cytolytic granules but have depleted cytolytic reservoirs due to more frequent degranulation. The CD56^dim^ cell with high levels of inhibitory signaling (IR^high^) due to increased receptor levels or upregulated HLA-C have a higher threshold of activation, and greater cytolytic reservoirs

The development and control of NK cell killing activity entails several distinct processes. Initially, there is an increase in the production of cytolytic effector molecules that accompanies the transition from CD56^bright^ to the CD56^dim^ stage. However, inhibitory signaling is required for the packaging of effector molecules into mature cytolytic granules (Goodridge et al. 2019). Finally, the activation and degranulation of the NK cell is controlled by the interplay of activating receptors detecting stressed cells and inhibitory signals recognizing self MHC. These processes and their impact on the functionality of NK cells are diagrammed in the model shown in Fig. 3. The immature CD56^bright^ NK cell is depicted as having low levels of effector molecules and lacking cytolytic granules, but still capable of cytokine secretion. The CD56^dim^ NK cells have a ten-fold higher level of effector molecules (Jacobs et al. 2001); however, their functional state is determined by the level of inhibitory signaling. The CD56^dim^, inhibitory receptor-negative (IR^−^) NK cell is hyporesponsive as it lacks the inhibitory signaling required to generate mature cytolytic granules. CD56^dim^ NK cells with a low level of inhibitory receptor signaling produce mature cytolytic granules, and thus represent the licensing/arming stage, or provision of ammunition for target cell killing. However, due to a lower level of inhibition, they are more prone to degranulation, resulting in a depleted cytolytic granule reservoir, and are subsequently less able to mediate serial killing. NK cells receiving a high level of inhibitory signaling due to either a greater number of inhibitory receptors, high affinity interactions, or upregulation of HLA-C by the NK cell are postulated to have increased *cis*-mediated inhibition, and would be less likely to be activated to kill. As such, they would be expected to maintain a larger cytolytic reservoir, enabling an increased serial killing capacity.

In immature NK cells, the lower levels of HLA-C as compared to mature cells may serve to calibrate their responsiveness in the context of the HLA-C expressed by the surrounding cells. It may be advantageous for a mature NK cell to express higher levels of HLA-C to increase *cis*-mediated inhibition, especially if it is in the circulation with more limited cell-cell contacts, thus increasing selectivity against potential targets. It has been shown that NK cells allowed to degranulate too frequently lose their killing potential, thus increasing their response threshold is beneficial for maintaining high levels of missing-self surveillance (Prager et al. 2019). Moreover, as educated NK cells are continually “tuned” by their environment, it seems useful for these cells to only respond against the strongest of signals, and as such, higher levels of *cis* HLA-C would limit killing of transiently stressed cells undergoing proliferation. The correlation between NK-Pro upregulation of HLA-C levels and the acquisition of lytic activity in mature CD56^dim^ NK cells suggests that this mechanism evolved to increase the activation threshold for lysis of targets to a higher level than that required for cytokine release by CD56^bright^ NK cells. This would be analogous to requiring further safety training for individuals that are licensed to carry lethal weapons.

## Data Availability

This review discusses published data only.
